# Investigation of Damage and Creep for Bedding’s Carbonaceous Slate with Chemical Erosion Effect

**DOI:** 10.3390/ma16145163

**Published:** 2023-07-22

**Authors:** Weihao Zeng, Zhenghong Chen, Yunpeng Xie, Qiunan Chen

**Affiliations:** 1 School of Civil Engineering, Hunan University of Science and Technology, Xiangtan 411201, China; 2 Research Center of Geotechnical and Structural Engineering, Shandong University, Jinan 250061, China

**Keywords:** carbonaceous slate, bedding angle, chemical erosion, creep characteristic

## Abstract

Tunnel projects in the southwestern mountainous area of China are in full swing. According to the tunnel burial depth, structural characteristics, and chemical erosion environments of the Lixiang railway tunnel, carbonaceous slate specimens obtained in the field were taken to experimentally investigate the physical, mechanical, and creep characteristics of the bedding’s slate specimens after chemical erosion. The results of scanning electron microscopy (SEM) indicate that chemical erosion leads to internal damage in the carbonaceous slate specimens, and the internal damages are increasing with the increase of erosion days. Moreover, the specimens’ ultrasonic test (UT) results prove that specimens with smaller bedding angles suffer a more serious erosion and induce more internal cracks. Under conventional triaxial compression conditions with 40 MPa of confining pressures, the conventional triaxial compressive strength (σ_s_) decreases with the decrease of the bedding angle and the increase of erosion days, and the failure modes of the specimens are mainly controlled by oblique shear fractures and accompanied by the occurrence of slip dislocation fractures between the bedding inclination. Under creep conditions with 40 MPa of confining pressures, the final deformations of specimens are increasing with the increase of erosion days, which means the longer the erosion days, the greater the deformations. The failure modes of the specimens under creep conditions are controlled by shear fractures, and for the specimen with a 60° bedding angle and long-term erosion, there are block separations and many cavities along the shear planes. Therefore, more attention should be paid to prevent serious failure of the surrounding rock if the surrounding rock has a bedding angle of 60° or suffers long-term erosion.

## 1. Introduction

Under a high in situ stress environment, rock masses present complex mechanical behaviors [1,2]; therefore, in engineering practice, a deep understanding of the mechanical behaviors of rock masses is the key to ensuring the safety and operation of deep-buried tunnels [3,4]. During long-time crustal movement, most sedimentary rocks and some metamorphic rocks have obvious bedding structures that will have a great influence on mechanical behaviors and result in discontinuity, heterogeneity, and anisotropy of the rock [5,6,7]. According to the engineering geological survey, the bedding structure is obvious in the surrounding rock mass of the Lixiang railway [8]. Since the bedding angle is varied and the mechanical properties are different, the influence of the bedding structures on the stability and safety of rock engineering cannot be ignored. Until now, the influences of bedding structures on the physical and mechanical properties of rock specimens have been extensively investigated. For example, He et al. [9] studied the rockburst ejection velocity of unloading sandstones with different bedding angles, and they found that the specimen with 60° bedding angles is the easiest one to rockburst, but the corresponding rockburst intensity is the weakest. Horizontal bedding is the least prone to rockburst, while the rockburst is strong once it occurs. Zhang et al. [10] investigated the effect of bedding on the dynamic compressive properties of slate; the results indicate bedding plays an increasingly dominant role in the failure of samples as the dip angle increases, and there is a strain rate decrease when the bedding angle increases from 30° to 60°. Liu et al. [11] conducted both quasi-static and dynamic uniaxial compression tests on bedding coal specimens, and they found that the bedding effect on the coal behavior on the quasi-static tests was more prominent than that on the dynamic tests. Hao et al. [12] investigated the influences of confining pressure and bedding angles on the mechanical behavior of brittle slate; they concluded that the fracture angle of slate presents a U-shaped curve with the bedding angle increases from 0° to 90°, and when the bedding angle is 45°–60°, the slate specimens present the lowest strength, and when bedding angle is below 15°, it shows the greatest strength. Wasantha et al. [13] carried out uniaxial compression tests on bedded sandstone with layers of different bedding orientations; they obtained that the failures of rock specimens with shallow bedding angles could be more violent and devastative than the failures of rock with steeply oriented bedding. Through Brazilian disc splitting tests, Liu et al. [14] concluded that the tensile strength of the bedding’s slate gradually decreases, with the bedding angle varying from 0° to 90°. Xia et al. [15] analyzed the influence of the geometrical properties of bedding planes on the direct shear strength characteristics, and they concluded two patterns of the shear fractures with a range of bedding plane geometries.

Except for the bedding structures, the chemical erosion environment is also encountered in the practical engineering of the Lixiang railway. Chemical erosion mainly affects the microstructure, particle adhesion, and mineral composition of the rock, which will change the rock’s physical and mechanical properties, creep properties, and macroscopic cracks [16,17,18]. There has been considerable research on rock behavior under chemical erosion environments for rock specimens. For example, Zhang et al. [19] conducted creep tests on cracked granite under various confining pressures and different chemical solutions, and they found that an acid solution has a more obvious influence on the creep behavior than that of an alkaline solution. Hu et al. [20] proposed constitutive models to describe the mechanical responses of cement-based material subjected to a long-term chemo-mechanical coupling effect. Using double-torsion load–relaxation tests, Callahan et al. [21] evaluated the effect of the chemical environment on the fracture toughness and subcritical fracture growth index (SCI) in silicified fault rocks; reductions in the SCI in all aqueous environments, with a >60% reduction in the alkaline solutions, were observed. To evaluate the effect of chemical solutions on the frictional properties of quartz-rich sandstone, triaxial compression tests have been performed on sandstone by Feucht and Logan [22]; the results suggest that the frictional resistance to sliding of the sandstone seems to be primarily controlled by the ionic strength of the solutions, with secondary control by the pH value of the solutions. Li et al. [23] investigated the micro-damage evolution and macro-mechanical property degradation of limestone due to chemical effects during triaxial compression conditions; they concluded that the reason for the mechanical property degradation of the rock is that the chemical solutions change the porosity and micro-damage evolution process.

The above studies indicate that bedding structures and chemical erosion can both effectively influence the mechanical properties of rock. It can be found that most of the existing studies only considered the single effect of the bedding structures or the chemical erosion on the mechanical characteristics of rock under short-term stress conditions. However, the coupling effect of the bedding structures and chemical erosion will significantly affect the long-term stability of surrounding rock for deep-buried tunnels, whereas related research is rare. To fill this gap, in this study, the coupling effects of bedding structures and chemical erosion on the characteristics of slate specimens under long-term creep conditions were investigated. Accordingly, the physical, mechanical, and creep characteristics of the bedding’s slate specimens after chemical erosion were obtained through the techniques of scanning electron microscopy (SEM), ultrasonic tests (UTs), conventional triaxial compression tests, and creep tests.

## 2. Experimental Setup

### 2.1. Test Apparatus

**Figure 1:**
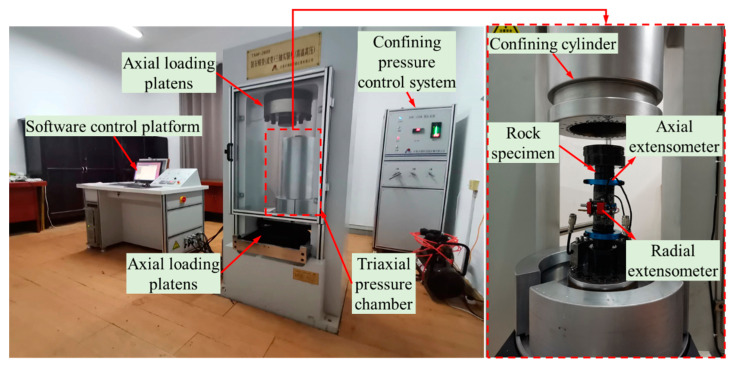
Photographs of the test apparatus.

The conventional triaxial compression tests and the triaxial creeping tests were performed on a servo-controlled rock mechanics testing system, TYJ-2000 (as shown in Figure 1), which consists of axial loading platens, a triaxial pressure chamber, a confining pressure control system, and a software control platform. The TYJ-2000 rock mechanics testing system can conduct conventional rock triaxial compression tests and creep tests under high temperatures and high pressures. The maximum axial load of the testing system is 2000 KN, and the maximum confining pressure is 100 MPa.

### 2.2. Rock Specimen Preparation

**Figure 2:**
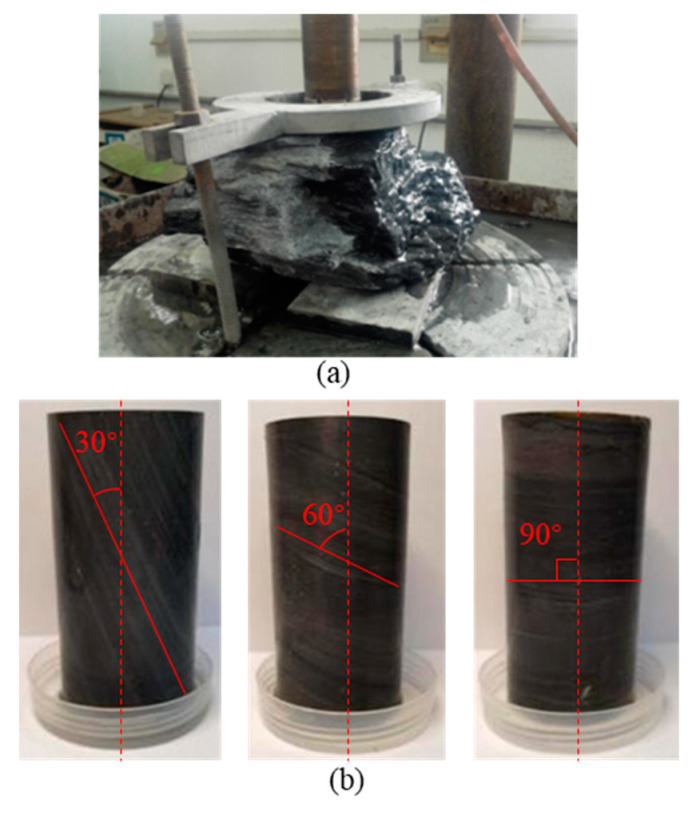
(**a**) Slate block; (**b**) specimens with different bedding angles.

Since the purpose of the research is to obtain the general damage and creep characteristics of the bedding’s carbonaceous slate under the chemical erosion effect, all the investigated specimens should be alike, except for the bedding angle and erosion days. Therefore, rather than collect rock cores in the drill holes to obtain testing specimens, the specimens were cored from an uneroded carbonaceous slate block, which is collected in a certain tunnel along the Lixiang railway. The carbonaceous slate block is gray (black) with no visible cracks, its lithology is compact, and the bedding structure is obvious. According to our previous research results of an X-ray diffraction (XRD) experiment, the mineral compositions and their contents of this carbonaceous slate are listed in Table 1 [24]. The rock specimens were cored from the slate block using the water drill method (Figure 2a). According to the geological investigations for the surrounding rock mass in the tunnel, the angle between the carbonaceous slate strata and the vertical direction is mostly around 25° to 90°. Therefore, three typical bedding angles of 30°, 60°, and 90° were investigated to capture the general law of the bedding angle effect. The angle between the drill bit direction and the horizontal bedding structure of the slate block is 30°, 60°, and 90°, respectively. To do so, standard cylinder specimens (50 mm × 100 mm) with three different bedding angles of 30°, 60°, and 90° were prepared (as shown in Figure 2b). The prepared specimens contained no visible damage or defects. Moreover, the end surfaces of the specimens were carefully polished, and the testing processes were well-designed to comply with the International Society of Rock Mechanics (ISRM) suggested testing requirements [25].

According to the water quality investigation results of a tunnel along the Lixiang railway, the underground water solution in this area holds a pH value of 4.8 to 5.3 and contains a variety of ionic components, mainly including Mg^2+^, Ca^2+^, SO_4_^2−^, and Cl^−^, indicating that the groundwater is a chemical erosion environment. To reproduce the chemical erosion effect of the SO_4_^2−^, Cl^−^, and H^+^ on the carbonaceous slate in this area, in this paper, dilute hydrochloric acid and CaCl_2_ and Na_2_SO_4_ solutions were used to prepare the chemical solvent with a pH value of 4.8.

### 2.3. Experimental Schemes

**Figure 3:**
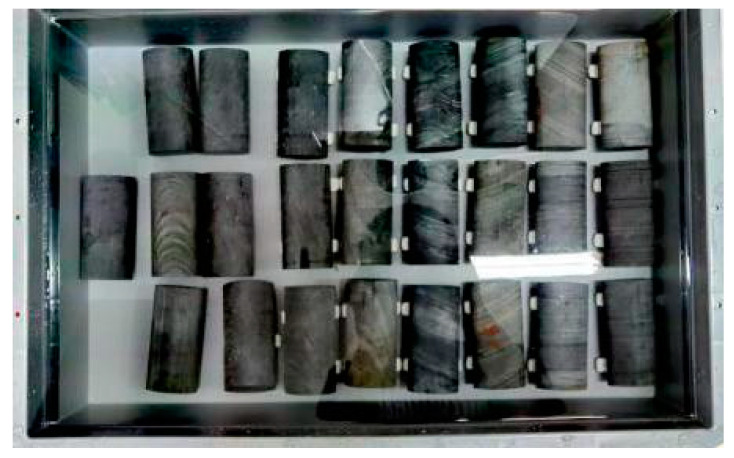
Photograph of the specimen’s erosion process.

The prepared specimens with three different bedding angles (30°, 60°, and 90°) were immersed in the prepared chemical solvent for 30 days’ erosion, 60 days’ erosion, and 90 days’ erosion, respectively (Figure 3). After the immersion, the natural dry specimens (0 days’ erosion) were taken as a reference, and conventional triaxial compression tests were carried out to obtain conventional mechanical properties. Moreover, triaxial creep tests were also conducted on specimens with three different bedding angles and four different erosion days to obtain creep characteristics. Accordingly, the specimens were divided into two groups: Group A for the conventional triaxial compression test and Group B for the triaxial creep test. The specific specimen grouping is shown in Table 2.

Considering the measured in situ stress in the field of the deep-buried Lixiang railway tunnel is 39.8 MPa, the confining pressure was set as 40 MPa in the conventional triaxial compression test and triaxial creep test.

**Figure 4:**
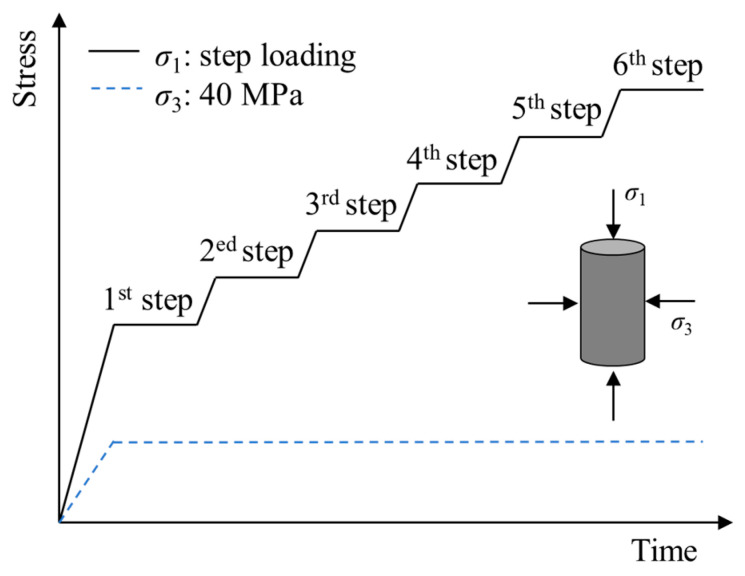
Stress paths for the triaxial creep test.

The specific experimental schemes in the conventional triaxial compression test and triaxial creep test were as follows:

(1) During the conventional triaxial compression tests, confining pressures were increased to 40 MPa at the rate of 0.10 MPa/s. The conventional triaxial compressive strength (*σ_s_*) for specimens could be obtained by increasing the axial loads at a rate of 0.20 MPa/s until the specimens were damaged;

(2) During the triaxial creep test, confining pressures were increased to 40 MPa at the rate of 0.10 MPa/s. The axial pressure was step-loaded at a rate of 0.20 MPa/s, the first step was loaded to 0.4 times the conventional triaxial compressive strength (*σ_s_*), and the subsequent load of each step increased by 0.1 times the *σ_s_* until creep failure occurred in the specimen (as shown in Figure 4).

## 3. Physical Properties of the Bedding’s Slate Specimens after Chemical Erosion

### 3.1. Microscopic Damage Properties of the Bedding’s Slate Specimens after Chemical Erosion

**Figure 5:**
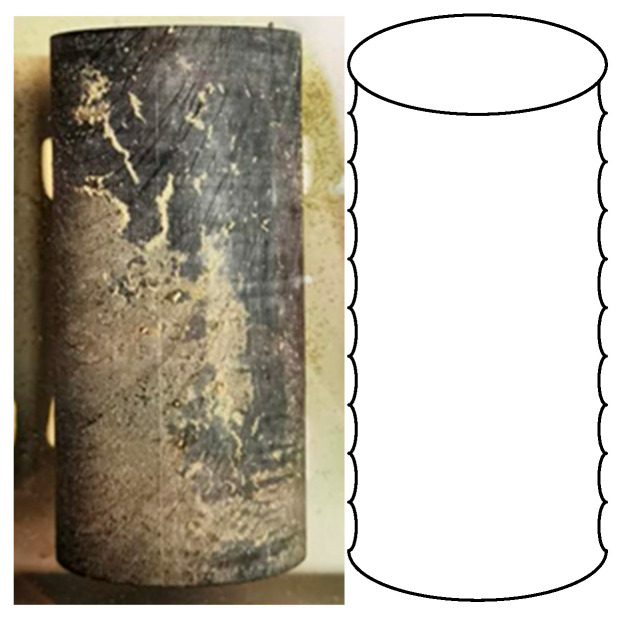
Photograph and schematic diagram of surface damage after chemical solvent erosion.

Affected by the high in situ stress in the southwest mountainous area, the microstructure of the collected slate specimens is dense and compact. However, there are still many microscopic cracks that cannot be detected by the eyes, especially for specimens after chemical erosion. As shown in Figure 5, the chemical solvent will have a significant influence on the microscopic damage of the slate specimens. Therefore, after different erosion days by a chemical solvent, the microstructure of the slate specimens will be damaged to different degrees. The microstructure damages have an important effect on the physical and mechanical properties of rock. To figure out the microstructure damage properties of the slate specimens by a chemical solvent, they were analyzed by a Gemini Sigma 300 scanning electron microscope, which is a widely used technology to obtain the micromorphology properties of objects [26,27].

**Figure 6:**
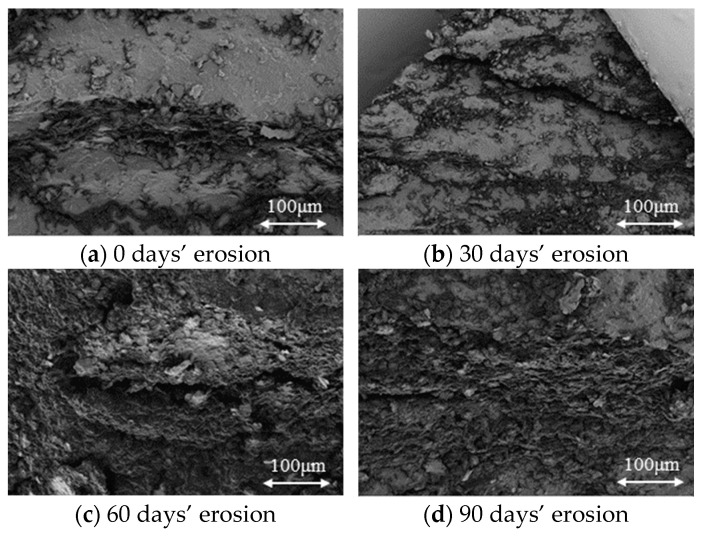
Typical SEM images of specimens under different erosion days.

To conduct SEM, 20 thin slices (no more than 2 cm in length and width) of slate specimens with four different erosion days were taken along the bedding plane. Typical SEM images of the specimens under different erosion days are shown in Figure 6. According to the images, it can be found that the specimens without erosion (0 days’ erosion) present a stepped structure, and the step surface is relatively flat and compact. For the specimens after 30 days’ erosion, there are many small and loose blocks on the surface of the stepped structure, and some micro-cracks emerged in the specimen slice. For the specimens after 60 days’ erosion, the edges and corners of the stepped structures are fuzzy and loose, showing a pit-like structure; moreover, the micro-cracks are the largest and most obvious among the four kinds of slices. For the specimens after 90 days’ erosion, the stepped structure is mostly a deep pit structure, and a lot of micro-cracks are filled or buried by loose and small blocks. Therefore, it can be concluded that the internal cracks induced by chemical erosion are increasing with the increase of erosion days.

### 3.2. Ultrasonic P-Wave Velocity of the Bedding’s Slate Specimens after Chemical Erosion

Longitudinal wave velocity in the ultrasonic tests (UTs) is one of the important parameters that can reflect the difference in the rock’s physical properties, such as the integrity, density, porosity, etc. [28,29] Therefore, by measuring the acoustic wave propagation velocity of the slate specimens, the integrity of the specimens can be analyzed (the internal cracks’ enrichment degree), and the relationship between the wave velocity and the bedding angle and chemical erosion degree can be explored.

In this paper, a ZBL-U5200 nonmetallic ultrasonic detector was used to detect the P-wave velocity of slate specimens with different bedding angles and chemical erosion days. During the detection, petroleum jelly was used as the coupling material between the rock specimens and transducer. According to the working principle of the nonmetallic ultrasonic detector, if there are more internal cracks or serious degradation in the specimen, the detected wave velocity of the specimen is lower [30,31].

**Figure 7:**
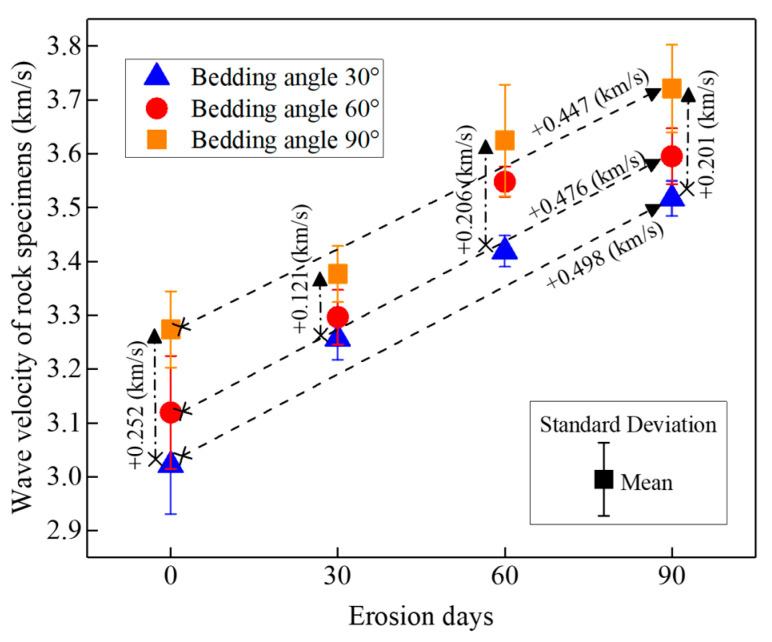
Wave velocity of specimens with different bedding angles with different erosion durations.

Figure 7 shows the wave velocity of specimens with different bedding angles and different erosion duration days. According to the descriptive statistics of three specimens under the same states (the same bedding angle and erosion days), it is found that the standard deviations of the wave velocity for the specimens are less than 0.1, which means the difference in the measured values is very small for specimens under the same states. Therefore, it is believed that the differences among the specimens under the same states are small.

Moreover, according to the analyses of the mean values for specimens under the same erosion days, the velocity of the ultrasonic longitudinal wave increases with the increase of the bedding angle. For example, for specimens under 30 days’ erosion, the increasing amount of wave velocity is 0.121 (km/s) when the bedding angle increased from 30° to 90°. This means that specimens with smaller bedding angles suffered serious erosion and induced more internal cracks. On the other hand, for specimens with the same bedding angles, it shows that the velocity of the ultrasonic longitudinal waves increases with the increase of erosion days. For example, for specimens with a 30° bedding angle, the increasing amount of the wave velocity is 0.498 (km/s) when the erosion days increased from 0 days to 90 days. The principal reason for this phenomenon is that the greater the erosion days, the more liquid will fill the microscopic cracks in the rock specimens. The effect of liquid filling on acoustic waves is greater than that of internal cracks induced by chemical erosion.

## 4. Conventional Triaxial Characteristics of the Bedding’s Slate Specimens after Chemical Erosion

According to the above analysis of the physical properties of the bedding’s slate specimens after chemical erosion, it is known that the chemical erosion changed the microstructures of the slate specimens; therefore, the macro-mechanical properties of the specimens will be changed as well. To investigate the compression characteristics of the bedding’s slate specimens after chemical erosion under 40 MPa of in situ stress conditions, conventional triaxial compression tests with 40 MPa of confining pressure were conducted on specimens with different bedding angles and erosion days.

### 4.1. Conventional Deformation and Triaxial Strength Characteristics

**Figure 8:**
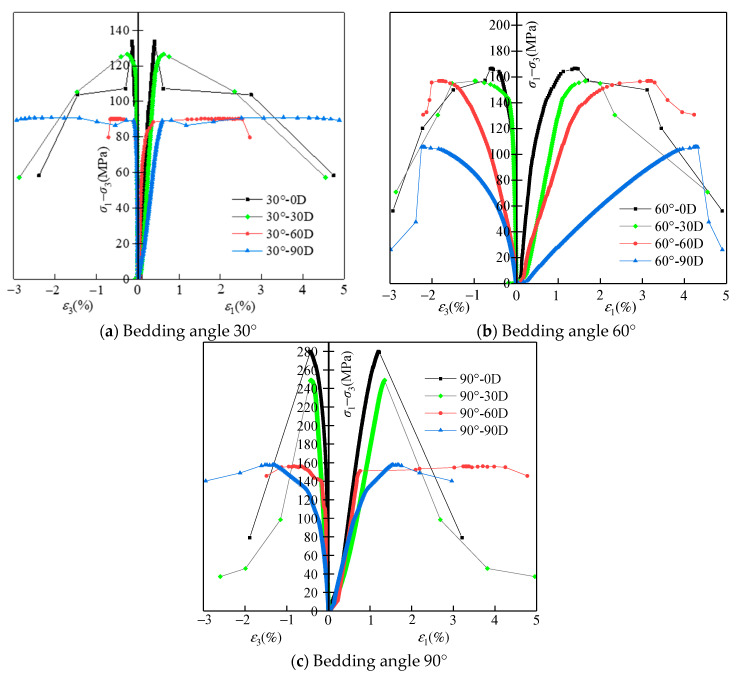
Stress–strain curves of specimens under triaxial compression tests.

The stress–strain curves of specimens under conventional triaxial compression tests are shown in Figure 8. Before the peak of deviation stress (*σ*_1_−*σ*_3_), the stress–strain curves increased linearly, indicating elastic deformations of the specimens. After the peak of the deviation stress, the stress–strain curves of the specimens with different bedding angles and erosion days are diverse. For the specimens with 30° and 90° bedding angles, under short-term erosions (0 days’ erosion and 30 days’ erosion), the specimens’ stress dropped sharply, with little strain increase, indicating a brittle failure, whereas, for the specimens with 30° and 90° bedding angles, under long-term erosions (60 days’ erosion and 90 days’ erosion), the specimens’ stress drop after failure is small, and there is a great strain increase, indicating ductile failure. The variations of stress–strain for the specimens with a 60° bedding angle are discrepancies from that of the specimens with 30° and 90° bedding angles. Under four different erosion days, the specimens with a 60° bedding angle all showed brittle failure features in the stress–strain curve. However, the specimens with a 60° bedding angle showed greater axial and radial strain at the peak of the deviation stresses than the specimens with 30° and 90° bedding angles, and the values of the axial and radial strain at the peak of the deviation stresses increased with the increase of erosion days. The reasons for this discrepancy phenomenon for the specimens with a 60° bedding angle can be interpreted by the fracture modes analysis in the next section.

**Figure 9:**
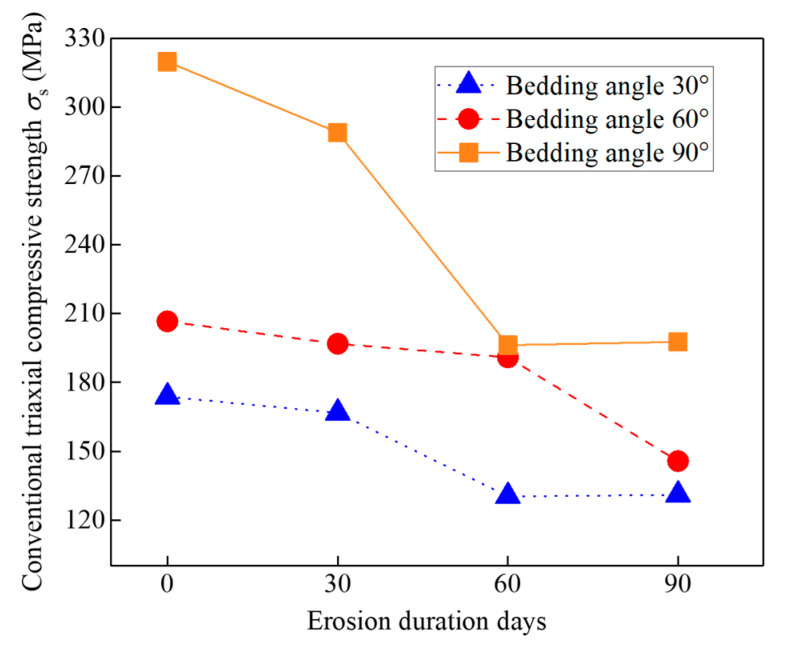
Conventional triaxial compressive strength with different bedding angles and erosion days.

Based on the stress–strain curves, the conventional triaxial compressive strength (*σ_s_*) of specimens can be obtained by adding 40 MPa of confining pressure to the peak of deviation stresses. Figure 9 shows the relationship of the *σ_s_* with the bedding angle and erosion days. For specimens under the same erosion days, the *σ_s_* decreased with the decrease of the bedding angle. This result can be explained by the variation of the wave velocity for the specimens with different bedding angles; in Section 3, it proves that the specimens with a smaller bedding angle exhibited a lower wave velocity because they suffered serious erosion and induced more internal cracks; more internal cracks in specimens will induce a lower *σ_s_*. On the other hand, for the specimens with the same bedding angle, the *σ_s_* decreased with the increase of erosion days. This conclusion is consistent with the results of the SEM images for the specimens under different erosion days, as shown in Figure 6, which indicates long-term erosion will induce more internal cracks and loose structures, and loose structures will result in lower *σ_s_*. Moreover, for the specimens with a 90° bedding angle under 0 and 30 days’ erosion, their *σ_s_* is greater than that of the other specimens; this is because they suffered slight erosion, and at the same time, the 90° bedding structure was vertical to the axial stress, which made them harder to break.

### 4.2. Conventional Triaxial Failure Modes

**Figure 10:**
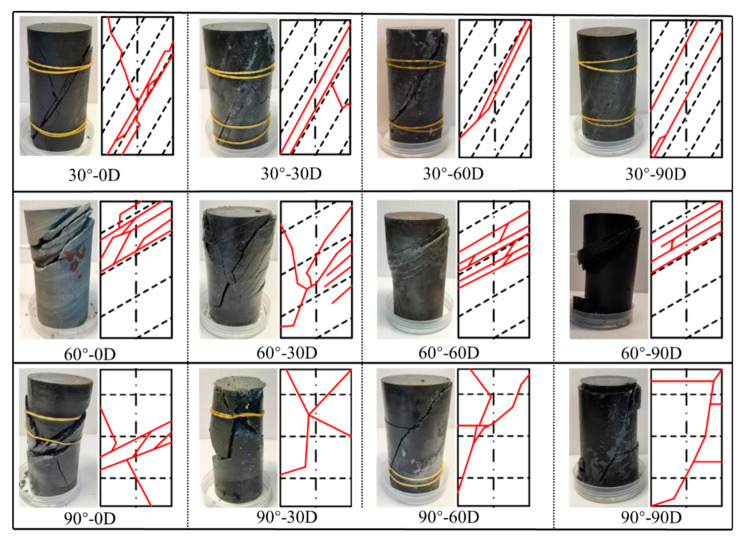
Failure modes and failure sketches of specimens under triaxial compression tests.

A deep understanding of the failure behavior of rock is important for the prediction and protection of engineering disasters. In this paper, the failure modes of specimens with different bedding angles and erosion days under conventional triaxial conditions were analyzed and compared. Figure 10 shows the failure modes and failure sketches of specimens under triaxial compression tests. It can be found that under conventional triaxial conditions, for specimens with 30° bedding angles, the main fractures in the specimens are connected diagonally along the bedding inclination, and X-shaped small secondary cracks are visible; moreover, with the increase of erosion days, the fractures along the bedding inclination are more obvious, and secondary cracks are rarer. For specimens with 60° bedding angles, there are several cut-through fractures propagated along the bedding inclination, resulting in spalling failure, which explains why the specimens with 60° bedding angles show greater axial and radial strain than that of the specimens with 30° and 90° bedding angles. Furthermore, with the increase of erosion days, the cut-through fractures changed from scattered to concentrated. For specimens with 90° bedding angles, the specimens under short-term erosion (0 days’ erosion and 30 days’ erosion) are relatively broken and show a common X-type conjugate inclined to plane shear failure, whereas the specimens’ failure under long-term erosion (60 days’ erosion and 90 days’ erosion) are controlled by shear failure, together with horizontal cracks.

In conclusion, the failure modes of specimens with different bedding angles and erosion days under conventional triaxial conditions are affected by the bedding’s inclination angle. The failure modes of the specimens are mainly controlled by oblique shear fractures and accompanied by the occurrence of slip dislocation fractures between the bedding inclination. Especially with increasing erosion days, the fracture development along the bedding’s inclination angle is gradually obvious, which indicates that chemical erosion accelerates the corrosion of the interlayer.

## 5. Creep Characteristics of the Bedding’s Slate Specimens after Chemical Erosion

Through the above analyses of the physical properties and triaxial compression test results, the basic physical and mechanical characteristics of the bedding’s slate specimens after chemical erosion are clear. In engineering practice, since the surrounding rock will deform greatly under high in situ stress and the deformation process will last for a long time, the long-term stability of the surrounding rock is the key to ensuring the safety and operation of deep-buried tunnels in high in situ stress environments, especially for the surrounding rock of the bedding’s slate subjected to chemical erosion. Therefore, in this section, to obtain the long-term deformation and failure characteristics, the creep tests were carried out on specimens with different bedding angles and chemical erosion days.

### 5.1. Creep Deformation Characteristics

(1) Influence of bedding angles on creep deformation characteristics

**Figure 11:**
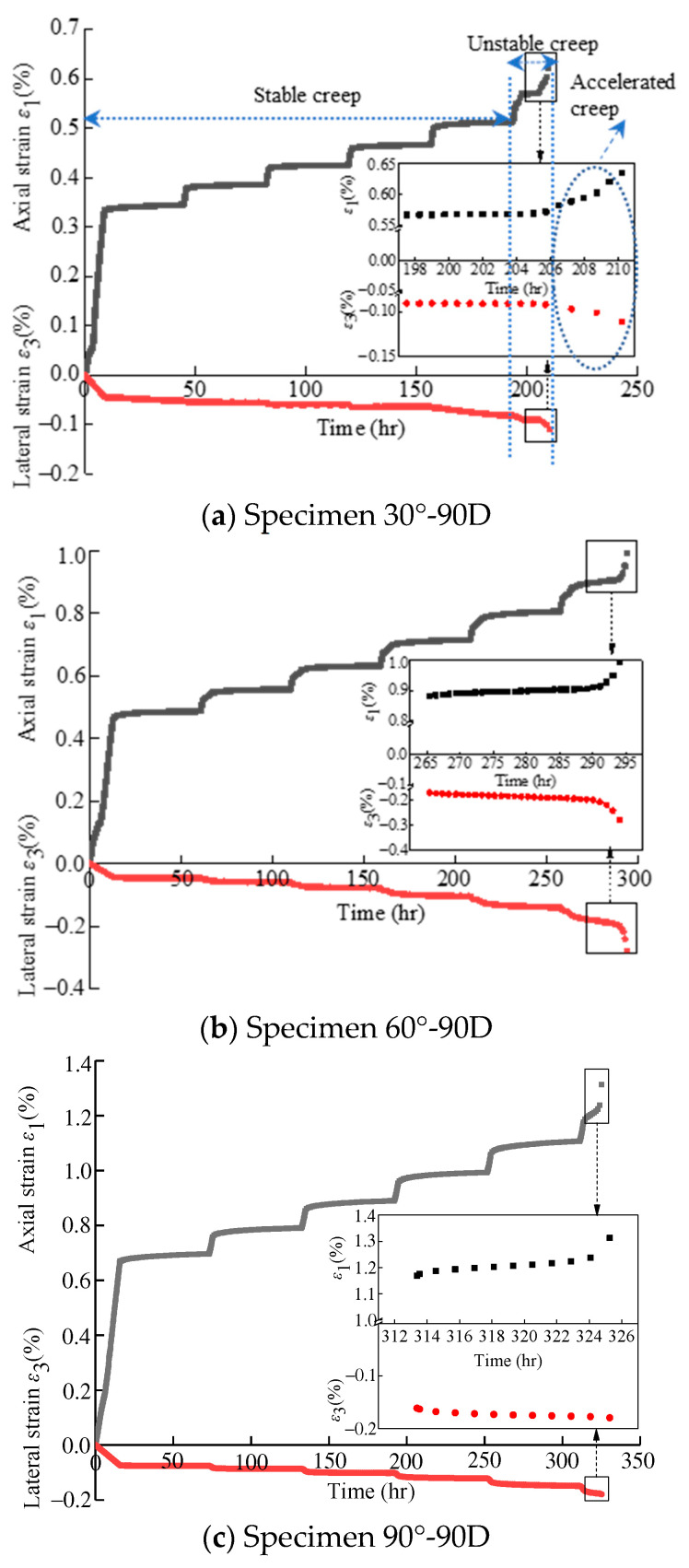

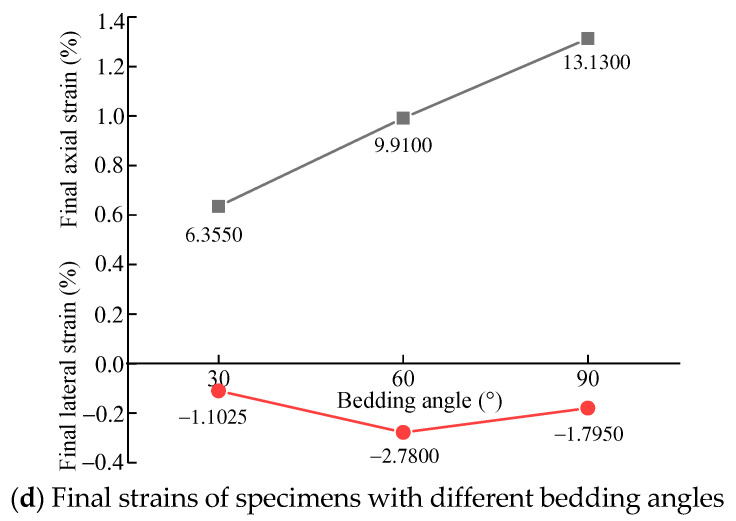
Creep curves and final strains of specimens with different bedding angles after 90 days of erosion.

According to the experimental scheme, the triaxial creep tests under a confining pressure of 40 MPa were carried out on the specimens with bedding angles of 30°, 60°, and 90° after 90 days of erosion. The strain–time curves of the creep tests for the specimens with different bedding angles after 90 days of erosion are plotted in Figure 11. It can be found that at the last step of loading, the creep of the specimen changed from a stable creep to an unstable creep, resulting in specimen failure. At the same time, an unstable accelerated creep occurred suddenly, indicating sudden brittle specimen failure.

Moreover, comparing the strain–time curves of specimens with bedding angles of 30°, 60°, and 90° after 90 days’ erosion, it is obvious that the axial deformations of accelerated creep stages increase with the increasing of the bedding angles; thus, the final axial strain increases (as shown in Figure 11d), whereas the lateral deformations of the accelerated creep stages are different. For the specimens of 30°-90D, the lateral deformations of the accelerated creep stage increase inconspicuously; for the specimens of 60°-90D, the lateral deformations of the accelerated creep stage increase obviously; for the specimens of 90°-90D, the lateral deformations show no accelerated creep stage. These variations of lateral deformations of the accelerated creep stage indicate the difference in the failure modes for specimens with different bedding angles under creep tests, which will be interpreted next in Section 5.2.

(2) Influence of erosion days on creep deformation characteristics

**Figure 12:**
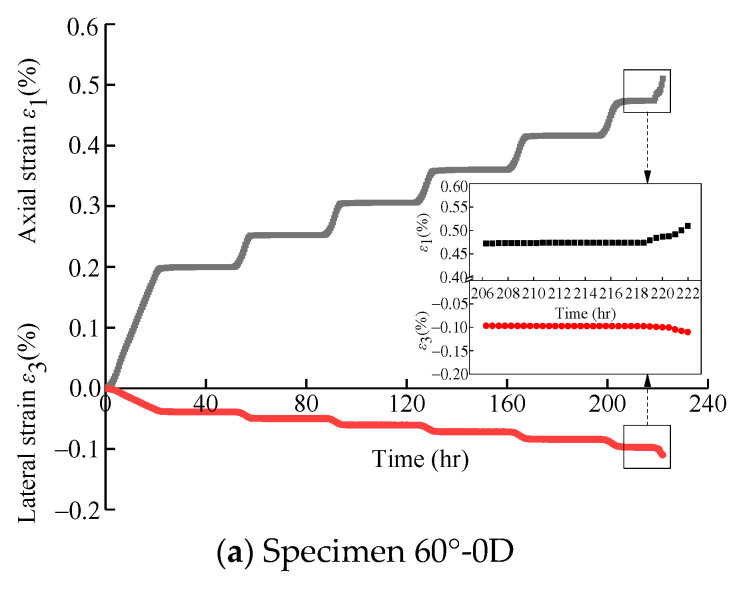

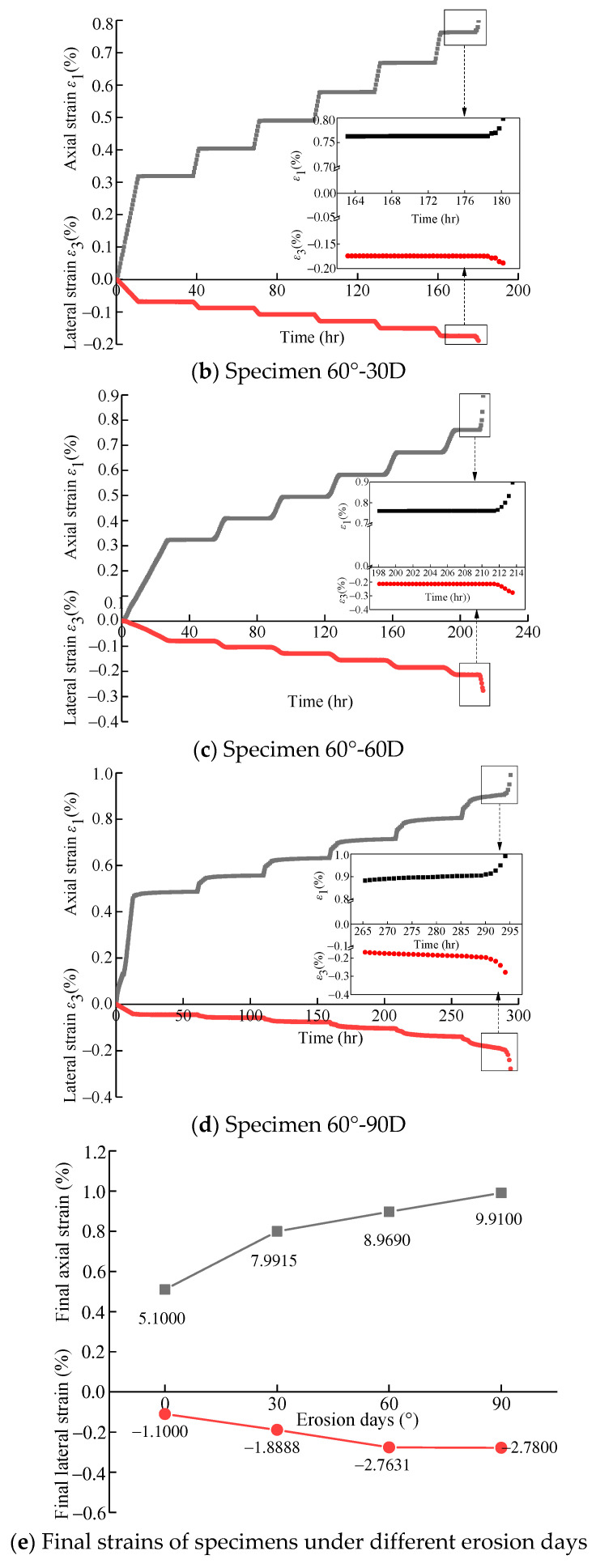
Creep curves of specimens with a bedding angle of 60° under different erosion days.

According to Section 4.1 and Section 5.1, there are discrepancies in the deformation characteristics for specimens with a 60° bedding angle under conventional triaxial tests and creep tests compared with 30° and 90° bedding angles. Thus, triaxial creep tests under a confining pressure of 40 MPa were carried out on the specimens with 60° bedding angles after 0, 30, 60, and 90 days of erosion. The strain–time curves of the creep tests for specimens with different erosion days are plotted in Figure 12. It can be found that for the specimens under long-term erosion (60°-60D and 60°-90D), the specimens’ unstable accelerated creep occurred more suddenly than that of the specimens under short-term erosion (60°-0D and 60°-30D), indicating a more sudden brittle specimen failure for the specimens under long-term erosion. Moreover, the final axial and final lateral deformations increase with the increase of erosion days (as shown in Figure 12e), which means the longer the erosion days, the greater the deformation.

### 5.2. Creep Failure Modes

**Figure 13:**
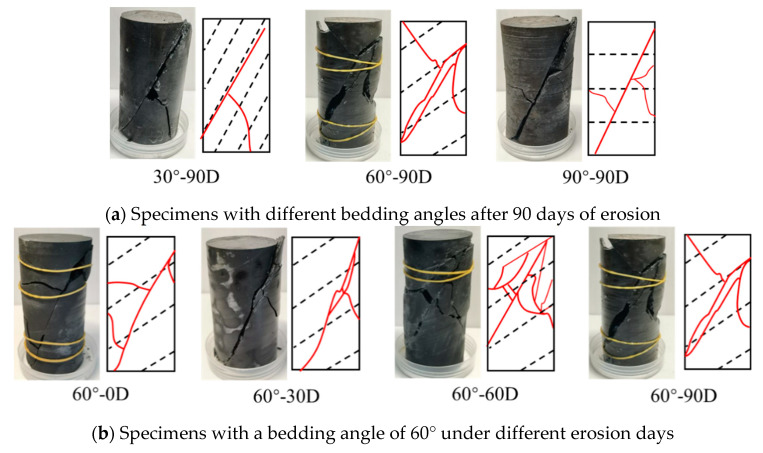
Failure modes of specimens under creep tests.

Analyzing the creep failure modes of the bedding’s slate subjected to chemical erosion can provide references for predicting failure behavior and the service state of engineering the surrounding rock. In this section, the creep failure modes of specimens with different bedding angles and erosion days under creep conditions were analyzed and compared. Figure 13 shows the failure modes and failure sketches of specimens under creep tests.

As shown in Figure 13a, the failure modes of the specimens with different bedding angles are controlled by shear fractures, and there are some cavities along the shear planes. For the specimens of 60°-90D, the cavities along the shear planes are more obvious, inducing a small block separation, which is interpreted as an obvious increase of lateral deformation for the specimens of 60°-90D observed in Section 5.1. Therefore, this proves that in engineering practice, for engineering the surrounding rock of the bedding’s slate under long-term chemical erosion conditions, more attention should be paid to prevent the surrounding rock’s failure if the bedding angle is 60°.

As shown in Figure 13b, the failure modes of the specimens with different erosion days are controlled by shear fractures. Moreover, for the specimens under long-term erosion (60°-60D and 60°-90D), the failure is more serious, with more cavities and secondary cracks than that of the specimens under short-term erosion (60°-0D and 60°-30D). Thus, preventing the long-term erosion of the bedding’s slate in engineering practice is meaningful for preventing serious damage to the surrounding rock under creep conditions.

## 6. Conclusions

In this paper, based on the experimental results, the influences of the coupling effect of the bedding structure and chemical erosion on the physical properties, conventional triaxial characteristics, and creep characteristics of carbonaceous slate specimens are analyzed for the first time. The following conclusions can be obtained:

(1) Chemical solutions containing SO_4_^2−^, Cl^−^, and H^+^ can damage the carbonaceous slate. According to the SEM results, the internal cracks and loose structures induced by chemical erosion are increasing with the increase of erosion days. Moreover, acoustic wave velocity results prove that specimens with smaller bedding angles suffer more serious erosion and induce more internal cracks;

(2) Under conventional triaxial compression conditions, for specimens with the same erosion days, the conventional triaxial compressive strength (*σ_s_*) decreases with the decrease of the bedding angle. On the other hand, for specimens with the same bedding angle, the *σ_s_* decreases with the increase of erosion days. The failure modes of the specimens are mainly controlled by oblique shear fractures and accompanied by the occurrence of slip dislocation fractures between the bedding inclination;

(3) Under creep conditions, for specimens with the same erosion days, the final axial deformations increase with the increase of the bedding angles, whereas the final lateral deformation increases obviously for the specimens with bedding angles of 60°. Moreover, the final axial and final lateral deformations increase with the increase of erosion days, which means the longer the erosion days, the greater the deformations. The failure modes of the specimens with different bedding angles and different erosion days are controlled by shear fractures, and for the specimens with 60° bedding angles and long-term erosion, there are block separations and many cavities along the shear planes.

These conclusive remarks can provide useful information for the safety and operations of deep-buried tunnels with the surrounding rock of the bedding’s carbonaceous slate. On the one hand, smaller bedding angles and longer erosion days will result in weakening the physical and mechanical properties. On the other hand, more attention should be paid to prevent serious failure and large deformation of the surrounding rock if the surrounding rock has a bedding angle of 60° or suffers long-term erosion. The drawback of the experiments herein is that the rock specimens were obtained from an integrated block rather than a filed borehole, which means the specimens were without any explicit natural fractures or flaws. However, in reality, the rock mass contains many natural fractures. Therefore, to further understand the damage and creep characteristics of carbonaceous slate rock masses, the effect of natural fractures should be involved, which will be considered in our follow-up studies.

## Figures and Tables

**Table 1:** Contents and structure characteristics of mineral composition [24].

Mineral Composition	Quartz	White Mica	Albite	Graphite
Mineral Content	72%	16%	9%	<3%
Structure characteristics	Granular or dense aggregate, without cleavage	Scaly or flaky aggregate, perfectly cleaved	Glassy crystal, medium cleavage	Carbon crystal, perfectly cleaved

**Table 2:** Specimens for the tests.

	Specimen Number	Erosion Days	Bedding Angle (°)
Conventional triaxial compression tests (Group A)	A-30°-0D	0	30°
	A-30°-30D	30	
	A-30°-60D	60	
	A-30°-90D	90	
	A-60°-0D	0	60°
	A-60°-30D	30	
	A-60°-60D	60	
	A-60°-90D	90	
	A-90°-0D	0	90°
	A-90°-30D	30	
	A-90°-60D	60	
	A-90°-90D	90	
Triaxial creeping tests (Group B)	B-30°-90D	90	30°
	B-60°-30D	30	60°
	B-60°-60D	60	
	B-60°-90D	90	
	B-90°-90D	90	90°

## Data Availability

Data sharing is not applicable to this article.
